# Prevalence of anti-SARS-CoV-2 antibodies in people attending the two main Goma markets in the eastern Democratic Republic of the Congo

**DOI:** 10.1017/S0950268823001498

**Published:** 2023-09-19

**Authors:** Prudence Ndeba Mitangala, Leonid M. Irenge, Edgar Tsongo Musubao, Jean Bosco Mbeva Kahindo, Patrick Ndeba Ayonga, Israël Kyembwa Safari, Janvier Bonane Kubuya, Edmon Namegabe Ntabe, Raphaël Kakongo Kabangwa Senga, Guy Ndongala Mutombo, Jérôme Ambroise, Jean-Luc Gala

**Affiliations:** 1 Université Catholique de Bukavu, Bukavu, Democratic Republic of Congo; 2 Université Officielle de Ruwenzori, Butembo, Democratic Republic of Congo; 3 Center for Applied Molecular Technologies, Institute of Clinical and Experimental Research, Université catholique de Louvain (UCLouvain), Woluwe-Saint-Lambert, Belgium; 4 Institut de Techniques Médicales, Goma, Democratic Republic of Congo; 5 Département des maladies infectieuses et tropicales, Université de Bordeaux, Bordeaux, France; 6 Inspection Provinciale de la Santé du Nord Kivu, Goma, Democratic Republic of Congo; 7 Division Provinciale de la Santé du Nord Kivu, Goma, Democratic Republic of Congo; 8 Université Libre des Pays des Grands Lacs, Goma, Democratic Republic of Congo; 9 Laboratoire Provincial Ami Labo Nord Kivu, Goma, Democratic Republic of Congo

**Keywords:** African paradox, city markets, COVID-19 infection, customers, DRC, SARS-CoV-2, seroprevalence, urban settlement, vendors

## Abstract

The Democratic Republic of the Congo (DRC) officially reports low coronavirus disease 19 (COVID-19) prevalence. This cross-sectional study, conducted between September and November 2021, assessed the COVID-19 seroprevalence in people attending Goma’s two largest markets, Kituku and Virunga. A similar study in a slum of Bukavu overlapped for 1 month using identical methods. COVID-19-unvaccinated participants (n = 796 including 454 vendors and 342 customers, 60% of whom were women) were surveyed. The median age of vendors and customers was 34.2 and 30.1 years, respectively. The crude and adjusted anti-SARS-CoV-2 antibody seroprevalence rates were 70.2% (95% CI 66.9–73.4%) and 98.8% (95% CI 94.1–100%), respectively, with no difference between vendors and customers. COVID-19 symptoms reported by survey participants in the previous 6 months were mild or absent in 58.9% and 41.1% of participants with anti-SARS-CoV-2 antibodies, respectively. No COVID-19-seropositive participants reported hospitalisation in the last 6 months. These findings are consistent with those reported in Bukavu. They confirm that SARS-CoV-2 spread without causing severe symptoms in densely populated settlements and markets and suggest that many COVID-19 cases went unreported. Based on these results, the relevance of an untargeted hypothetical vaccination programme in these communities should be questioned.

## Introduction

Coronavirus disease 2019 (COVID-19), a pandemic that swept the world at the end of 2019, is caused by severe acute respiratory syndrome coronavirus-2 (SARS-CoV-2), an RNA virus beta-CoV of group 2B virus that was originally described in the Chinese province of Hubei [1]. The pandemic has had a serious impact on global health and the world economy. Two years after the start of the COVID-19 pandemic and despite alarming predictions about its spread and major impact on the health and mortality of local populations in Africa [2–5], the resilience of sub-Saharan African countries has been much better than expected. Several reports support this observation by highlighting the low number of severe cases and deaths caused by COVID-19 in sub-Saharan Africa over the last 2 years [5–9]. Several authors have referred to the phenomenon as the ‘African paradox’, advocating factors such as demographics, climate and environmental factors, under-reporting, cross-immunity, or community-based responses as explanations [10, 11].

The Democratic Republic of the Congo (DRC), which reported its first COVID-19 case on 10 March 2020 [12], is one of the countries that have so far reported a rather low number of COVID-19 cases. As of 1 January 2023, this 90 million-person country had reported 95 257 COVID-19 cases and 1460 deaths directly attributed to COVID-19 [13]. North Kivu province, which borders Uganda and Rwanda, reported its first COVID-19 case on 30 March 2020 [14]. It has since confirmed a total of 11 007 cases and 603 deaths related to COVID-19 [14] for a population of around 9 million inhabitants [15]. These low figures reported for North Kivu province, as well as for the entire DRC, are intriguing, given the virtual absence of measures to contain the spread of SARS-CoV-2 in the country’s cities and towns. In addition, the DRC has one of the lowest vaccination rates in the world, with less than 3% of the population fully vaccinated at the time of the survey [16]. In the city of Goma, the current vaccination rate was very low, with only 0.14% having been completely vaccinated [17].

The country’s capacity to identify SARS-CoV-2 by real-time PCR is very limited and does not fulfil the World Health Organization guidelines for SARS-CoV-2 screening at the national level [18]. This shortcoming is one of the reasons suggesting some under-reporting of COVID-19 cases and deaths in the DRC. It has also been proposed that the population in developing countries had a stronger protective immunity because of a variety of recurring infectious diseases, among which malaria [19–22] and endemic coronavirus infections [23], although this is still subject to debate [24]. Indeed, the low proportion of elderly individuals characterising the demographic structure of sub-Saharan African countries is advocated in some reports as the main reason for the low COVID-19 mortality observed in these countries [22]. This hypothesis would, at least partially, explain the low demand of medical care and COVID-19-related death rate compared to other continents and contribute to the under-reporting of cases. Moreover, the scarcity of epidemiological surveys on the prevalence of COVID-19 in the country drastically hinders the assessment of the true scale of the COVID-19 pandemic in the country. It should be noted that serological analyses in this country with 90 million inhabitants are only available in the cities of Kinshasa and Bukavu and that these seroprevalence data suggest the spread of SARS-CoV-2, resulting in anti-SARS-CoV-2 antibodies in the majority of survey participants [6, 25].

Bordered to the north by Lake Kivu, Goma is one of the largest and busiest cities in the DRC. It is an important communication hub connecting Rwanda, Uganda, Kenya, Kinshasa, Bukavu, and Bujumbura as well as the DRC’s countryside west of Lake Kivu, which includes the provinces of North Kivu and South Kivu. Given its location and connections, Goma is a likely hotspot for the rapid expansion of COVID-19 among its population and beyond.

The purpose of this survey was to assess the seroprevalence of anti-SARS-CoV-2 antibodies in the crowded markets of Kituku and Virunga of the city of Goma and to compare the seroprevalence of anti-SARS-CoV-2 in the densely populated settlement of Kadutu in Bukavu [6]. Bukavu and Goma surveys were conducted 3 months apart, from June to September 2021 and September to November 2021, respectively. This was conducted to avoid major differences due to the pandemic’s temporal evolution.

## Material and methods

Between 1 September 2021 and 8 November 2021, we conducted a survey to determine the seroprevalence of anti-SARS-CoV-2 antibodies among a sample of vendors and customers in the Kituku and Virunga markets of Goma, DRC. Goma, a city with about 1 million inhabitants, is located on the northern shore of Lake Kivu, straddling the Rwandan border. It is a major commercial hub in the country’s eastern region and home to several markets, notably the Virunga market (northeast of Goma) and Kituku market (west of Goma). At midday, the number of people present at Kituku and Virunga markets is around 20 000 in each, with approximatively 4000 vendors in each market.

The sample size of vendors to be enrolled in the study was calculated using sample size calculator software [26], based on the following parameters: i) a confidence level of 95%, a prevalence (population proportion) of 50%, a margin of error of 5%, and a target population of 30 000. The sample size was increased by 20% to account for non-consenting or absent vendors on the day of sampling, resulting in a total of 456, with a minimum of 380 participants.

## Data collection

A team of 7 members (i.e. outreach members, interviewers, and a laboratory technician) was sent to the two markets to carry out sampling and collect questionnaires. Following consent of market managers, a systematic random sampling of one out of every ten sellers in each row of the market was carried out. Market customers were recruited through convenience sampling and enrolled in the study at the same time. This sampling entailed enrolling each customer who agreed to participate and was present at a surveyed vendor stand. Firstly, as in a parallel study [6], selected participants (i.e. vendors and customers) were interviewed to assess their COVID-19 disease knowledge (i.e. transmission route of SARS-CoV-2, COVID-19, knowledge and compliance to non-pharmaceutical mitigation measures, whether at the market or at home), COVID-19 vaccination status, and whether they recalled having experienced COVID-19-related symptoms over the previous 6 months. The interviews were conducted by using a questionnaire. Non-pharmaceutical interventions (NPIs) to slow the spread of COVID-19 in markets were also considered by the investigators. Those included the installation of chlorinated water tanks for hand washing and the wearing of masks at the time of the interview. A mask was defined as a protective covering for the chin, mouth, and nose. Subsequently, fresh finger-prick blood was taken from each participant and placed directly into the well of the QuickZen COVID-19 IgM/IgG kit (ZenTech, Angleur, Belgium), an immune colloidal gold lateral flow test kit that detects IgM and IgG against SARS-CoV-2 S-RBD (receptor-binding domain of the S protein of SARS-CoV-2). The results were interpreted according to the manufacturer’s recommendations. In a parallel survey carried out in Bukavu in the same time period, 49 pre-COVID-19 serum samples that were collected in the district of Kadutu between April 2004 and May 2005 tested negative with the QuickZen assay [6]. The same assay was also tested in this study on an additional batch of 32 pre-COVID-19 plasma samples from the Goma population, which were collected in 2019 and stored at the AMI-LABO facility in Goma city. The Division Provinciale de la Santé du Nord-Kivu provided data on COVID-19 vaccination in North Kivu province [17].

## Statistical analyses

Statistical analyses were performed using the SPSS statistical package for Windows, version 26.0 (SPSS, Inc., Chicago, IL). The crude seroprevalence was calculated as the proportion of participants positive for anti-SARS-CoV-2 antibodies. The adjusted seroprevalence was calculated using the standard correction formula mentioned in a previous study by Sempos and Tian [27]: Adjusted Prevalence = (Crude prevalence + specificity - 1)/ sensitivity + specificity - 1).

Montesinos et al. previously calculated the combined IgG/IgM sensitivity and specificity of the QuickZen COVID-19 IgM/IgG kit to be 71.1% and 100.0%, respectively [28]. Differences in group proportions and categorical variables were assessed using the chi-square test. Odds ratios (ORs) for the presence of symptoms associated with COVID-19 in the presence or absence of anti-SARS-CoV-2 antibodies were calculated. A *P* value <0.05 was considered statistically significant. Adjusted *P* values were computed in R v.4.1.1 using the method of Benjamini et al. [29].

## Ethical considerations

The Université Catholique de Bukavu’s Internal Review Board (UCB/CIES/NC/022/2021) reviewed and approved this study. Before enrolment and sample collection by local first-line responders, all participants provided their consent, but given the low level of literacy of market attendees, only verbal consent was requested and recorded. Healthcare workers and physicians signed the following statement: ‘We have explained the study to the participants and are satisfied that he/she understands and consents to participate in the survey’.

## Results

In total, 796 participants (i.e. 454 vendors and 342 market customers) were included in the survey. Table 1 compares the socio-demographic characteristics of vendors and customers. The median age of vendors was 34.2, and vendors were younger than 40 years constituted 80.6% of all vendors. Customers were younger, with the median in this group of 30.1 years, and customers younger than 40 years constituted 87.7% of all customers interviewed.

**Table 1.** Socio-demographic characteristics and SARS-CoV-2 serology (IgG and/or IgM) of vendors and customers surveyed in two Goma markets from September to November 2021

				SARS-CoV-2 serology (crude seroprevalence)					
				Vendors			Customers		
	Study group			Positive	Negative		Positive	Negative	
Variable	Vendor *n* = 454 (%)	Customer *n* = 342 (%)	*P* value	*n* = 319 (70.3%)	*n* = 135 (29.7%)	*P* value	*n* = 240 (70.2%)	*n* = 102 (29.8%)	*P* value
Goma’s markets									
Kituku	218 (48)	163 (47.7)	0.97	152 (69.7)	66 (30.3)	0.81	112 (68.7)	51 (31.3)	0.57
Virunga	236 (52)	179 (52.3)		167 (70.8)	69 (29.2)		128 (71.5)	51 (28.5)	
Age (years)									
18–25	144 (31.7)	156 (45.6)	<0.01	99 (68.8)	45 (31.2)	0.81	108 (69.2)	48 (30.8)	0.94
26–40	222 (48.9)	144 (42.1)		156 (70.3)	66 (29.7)		102 (70.8)	42 (29.2)	
≥40	88 (19.4)	42 (12.3)		64 (72.2)	24 (27.8)		30 (71.4)	12 (28.6)	
Sex									
Female	296 (65.2)	179 (52.3)	<0.01	210 (70.9)	86 (29.1)	0.66	127 (70.9)	52 (29.1)	0.74
Male	158 (34.8)	163 (47.7)		109 (69.0)	49 (31.0)		113 (69.3)	50 (30.7)	
Interviewees wearing a mask									
Yes	80 (17.6)	85 (24.9)	0.01	55 (68.8)	25 (31.2)	0.74	59 (69.4)	26 (30.6)	0.86
No	374 (82.4)	257 (75.1)		264 (70.6)	110 (29.4)		181 (70.4)	76 (29.6)	
Residential district									
Goma	173 (38.1)	139 (40.6)	0.04	122 (70.5)	51 (29.5)	0.88	98 (70.5)	41 (29.5)	0.95
Karisimbi	267 (58.8)	181 (52.9)		188 (70.4)	79 (29.6)		126 (69.6)	55 (30.4)	
Nyiragongo	14 (3.1)	22 (6.4)		9 (64.3)	5 (35.7)		16 (72.7)	6 (27.3)	

In total, out of 796, 559 participants tested positive for IgM and/or IgG anti-SARS-CoV-2 antibodies, resulting in an overall crude seroprevalence of 70.2% (95% CI: 66.9%–73.4%). The adjusted seroprevalence of anti-SARS-CoV-2 antibodies was 98.8% (95% CI 94.1–100%). There was no significant difference between vendors (70.3%, 95% CI 66.1–74.5%) and customers (70.2%, 95% CI 65.0–75.0%) (*P* value = 0.98). The antibody distribution of anti-SARS-CoV-2 antibodies among positive participants was as follows: IgM 13.4%, IgG 53.4%, and both IgM and IgG 33.2%. The overall seroprevalence of anti-SARS-CoV-2 IgM antibodies was 46.6%, indicating recent SARS-CoV-2 infection and an active virus circulation in the weeks preceding the survey. The pre-pandemic sera (n = 32) from Goma were negative for anti-SARS-CoV2 antibodies, as were the 49 pre-pandemic sera from Bukavu [6]. There were no significant differences in seroprevalence rates between all groups analysed (vendors vs customers, women vs men, Kituku market participants vs Virunga market participants). Similarly, no significant differences in seroprevalence were found in age groups.

Both markets were overcrowded, with no social measures implemented to prevent the SARS-CoV-2 from spreading among market attendees. In terms of NPI, both markets had chlorinated water tanks for hand washing. Only 17.6% of the surveyed vendors wore a mask during the interview, whereas this rate was significantly higher among customers (24.9%) (*P* value = 0.01).

According to their anti-SARS-CoV-2 status, symptoms experienced by the group of vendors in the 6-month period preceding the survey are summarised in Table 2. Compared with participants who tested negative for the presence of anti-SARS-CoV-2 antibodies (n = 135), those who tested positive (n = 319) were more symptomatic, with odds ratios (ORs) consistently higher than 2.0 (adjusted *P* value <0.05) for each symptom with the exception of ageusia (OR = 1.0 adjusted *P* value = 0.15). In the group of vendors, 41.1% of participants with anti-SARS-CoV-2 antibodies did not recall having experienced any COVID-19-related symptoms (OR = 1.0; CI 95% 0.6–1.5%).

**Table 2.** Clinical symptoms and odds ratios among vendors surveyed in Kituku and Virunga Goma markets from September to November 2021

	SARS-CoV-2 serology			
Symptom	Positive *n* = 319 (%)	Negative *n* = 135 (%)	Odds ratio (CI 95%)	Adjusted *P* value
Fever	118 (37.0)	12 (8.9)	6.0 (3.2–11.4)	<0.01
Coughing	123 (38.6)	19 (14.3)	3.8 (2.2–6.5)	<0.01
Unexplained tiredness	86 (27.0)	8 (5.9)	5.9 (2.8–12.5)	<0.01
Headaches	30 (9.4)	3 (2.2)	4.6 (1.4–15.2)	0.02
Muscle pain	62 (19.4)	3 (2.2)	10.6 (3.3–34.5)	<0.01
Sore throat	41 (12.9)	3 (2.2)	6.5 (2.0–21.3)	<0.01
Anosmia	29 (9.1)	4 (3.0)	3.3 (1.1–9.5)	0.04
Ageusia	11 (3.4)	9 (0.7)	4.8 (0.6–37.4)	0.15
Fever and coughing	67 (21.0)	15 (11.1)	2.1 (1.2–3.9)	0.02
Fever, coughing, and muscle pain	59 (18.5)	9 (0.7)	30.4 (4.2–221.9)	<0.01
Absence of symptoms	131 (41.1)	80 (59.3)	1.0 (0.6–1.5)	1.0

## Discussion

From 1 September to 8 November 2021, we conducted a seroprevalence survey of anti-SARS-CoV-2 antibodies among vendors and customers in Kituku and Virunga, Goma’s two main markets. The results showed that unadjusted seroprevalence rates among vendors and customers were 70.3% and 70.2%, respectively. The participants’ age or gender did not influence the seroprevalence of anti-SARS-CoV-2 antibodies. The very low proportion of vendors (17.6%) and customers (24.8%) wearing a protective mask correctly is an indication of the weak implementation of preventive measures against the spread of COVID-19, or lack thereof, in these overcrowded settings where no NPI enforcement measures were visible. This is worsened by the fact that Goma sellers constantly advertise their wares by shouting, which facilitates virus transmission to market customers via aerosolisation [30].

While this survey’s seroprevalence rates are among the highest documented worldwide in an unvaccinated population, they are comparable to similar studies in other sub-Saharan African countries. In early 2022 in neighbouring Uganda, Briggs et al. found a seroprevalence rate of anti-SARS-CoV-2 antibodies above 80% in urban and rural areas, in unvaccinated, previously seronegative individuals, highlighting the very high SARS-CoV-2 infection rates, despite low case ascertainment. This high rate of seroconversion was attributed by the authors to the Omicron wave [8, 29]. According to a recent review of SARS-CoV-2 studies in Africa, anti-SARS-CoV-2 seroprevalence increased from 3.0% in April–June 2020 to 65.1% in July–September 2021 [31]. This later rate, obtained almost concurrently with our survey, is comparable to seroprevalence rates in Goma markets and Bukavu slum [6]. Although these rates cannot be directly extrapolated to the entire city of Goma, the concordance of high seroprevalence rates in the market vendors and customers groups (even though customers were not randomly sampled), suggests a high rate of SARS-CoV-2 transmission in the city.

Our study had several limitations. Firstly, Montesinos et al. determined the specificity and sensitivity of the assay based on 128 COVID-19-negative sera from healthy Belgian volunteers with no recorded contact with SARS-CoV-2 and 72 COVID-19-positive sera from Belgian patients who had displayed a positive SARS-CoV-2 signal [28]. In the absence of a multicentric validation of the assay and due to the high number of SARS-CoV-2 variants over the course of this pandemic, the adjustment of African seroprevalence rates based on the sensitivity and the specificity from the Montesinos et al. study [28] appears to be a potential source of bias. However, all pre-pandemic COVID-19 sera tested negative in Bukavu (n = 49) [6] and Goma (n = 32), confirming the 100% specificity of the test. Secondly, due to the design of this study and the country’s insufficient testing capacity, our serological data could not be compared to reverse transcription polymerase chain reaction (RT-PCR) results. Consequently, we were unable to determine whether SARS-CoV-2 transmission was still active in market vendors and customers at the time of the survey, despite the fact that the high rate of IgM indicates recent SARS-CoV-2 transmission in people accessing Goma markets. As a result, we were unable to determine the case-to-undetected infection ratio (CIR) among survey participants due to the lack of RT-PCR results. The DRC health system also lacked trustworthy databases and age-specific population data, making it impossible to calculate the infection fatality rate or adjust seroprevalence for age. Thirdly, the use of a qualitative anti-SARS-CoV-2 antibody test does not allow the assessment of the amount of antibody required to confer protective immunity against reinfection. Fourthly, we surveyed a cohort of vendors, which does not reflect the actual composition of households in Goma. Indeed, children, working-class people, and the elderly are not, a priori, represented in this group, where there is an over-representation of women. Finally, customers were selected using a convenience sampling method, which is not a reliable sampling method. Despite these potential biases, the high seroprevalence in this group gives a glimpse of the extent to which SARS-COV-2 might have circulated in the city of Goma, with markets serving as important nodes for its dissemination. While the relatively young age of the surveyed cohort contributes to the low COVID-19 morbidity, these results must be interpreted in the light of the previously discussed ‘African paradox’ and the hypothesis that recurring infectious diseases, among which malaria and other endemic coronavirus infections, may help increase protective immunity [6, 9–11, 19–22]. The observation that a large majority of the population examined in Bukavu [6] and Goma had been infected with COVID-19 without experiencing obvious damage suggests a protective role of the immunity attributed to recurring infectious disease, in line with other reports [20, 21]. This pattern may result from the demographic structure of the sub-Saharan African population [22], where mortality linked to prevalent infectious diseases, such as malaria, tends to be higher among vulnerable groups – including children younger than 5 years and the elderly – than among young adults [32]. Notably, weaker health systems, precarious livelihoods, poverty, and urban overcrowding all contribute to shaping this demographic structure [9]. Interestingly, these factors are also widely regarded as risk factors exacerbating the COVID-19 morbidity and mortality. While these factors would seemingly disadvantage the sub-Saharan population [7, 10, 11], a higher COVID-19 burden is observed within corresponding age groups in Italy [33–35], China [35–37], and other developing countries facing the same pandemic [38] [7, 10, 11, 34–38]. The intriguing hypothesis of acquired protective immunity unrelated to vaccination in the DRC’s COVID-19 population, based on this paradox, along with a very low capacity to detect the presence of the SARS-CoV-2 both in local communities and large cities, may contribute to the global underestimation of SARS-CoV-2 seroprevalence and COVID-19 cases in the official reports from DRC [25]. The low morbidity in our study contrasts somewhat with provincial data which recorded 603 COVID-19 related deaths in the province since March 2020, making North Kivu province the second worst COVID-19 affected province after Kinshasa [38]. One plausible explanation is that vulnerable people such as the elderly and immunocompromised individuals were the most severely affected by COVID-19, and this probably resulted in most COVID-19-related case fatalities. Therefore, if the current national COVID-19 vaccination rate (below 3%) can be used as an indicator of the weakness of the vaccination campaign in the country at the time of the survey, DRC policy makers should develop a workable policy focusing on protecting the vulnerable people through targeted vaccination, rather than continuing with an ineffective vaccination campaign that has obviously failed to reach a two-digits threshold.

In conclusion, our study found a high seroprevalence of anti-SARS-CoV-2 antibodies among market vendors and market customers in Goma, DRC. Despite a complete lack of vaccination against SARS-CoV-2, there was no significant morbidity reported in this cohort. This low health impact cannot be attributed to the country’s official policy for combatting the COVID-19 pandemic, but rather to the population’s enhanced capacity for acquired immunity to infectious diseases, including COVID-19, and to its youth. Our results confirm and strengthen those obtained in the same period of time in a slum of Bukavu [6] and question the relevance of vaccination in these communities. However, data from the countryside are necessary to draw a more thorough epidemiological map of COVID-19 in the DRC and to assess the potential health impact on other vulnerable populations.

## Data Availability

The databases used and/or analysed during the current study are available from the corresponding author upon request.
